# Relevance of Porcine Stroke Models to Bridge the Gap from Pre-Clinical Findings to Clinical Implementation

**DOI:** 10.3390/ijms21186568

**Published:** 2020-09-08

**Authors:** Marc Melià-Sorolla, Carlos Castaño, Núria DeGregorio-Rocasolano, Luis Rodríguez-Esparragoza, Antoni Dávalos, Octavi Martí-Sistac, Teresa Gasull

**Affiliations:** 1 Cellular and Molecular Neurobiology Research Group, Department of Neurosciences, Germans Trias i Pujol Research Institute, 08916 Badalona, Catalonia, Spain; mmelia@igtp.cat (M.M.-S.); ndgregorio@igtp.cat (N.D.-R.); 2 Neurointerventional Radiology Unit, Department of Neurosciences, Hospital Germans Trias i Pujol, 08916 Badalona, Catalonia, Spain; ccastanod@gmail.com; 3 Stroke Unit, Department of Neurology, Hospital Germans Trias i Pujol, 08916 Badalona, Catalonia, Spain; luisale555@hotmail.com (L.R.-E.); adavalos.germanstrias@gencat.cat (A.D.); 4 Department of Cellular Biology, Physiology and Immunology, Universitat Autònoma de Barcelona, 08916 Bellaterra, Catalonia, Spain; 5 Fundació Institut d’Investigació en Ciències de la Salut Germans Trias i Pujol (IGTP), Carretera del Canyet, Camí de les Escoles s/n, Edifici Mar, 08916 Badalona, Catalonia, Spain

**Keywords:** stroke, animal models, pig, swine, gyrencephalic brain, white matter damage, connectivity, translational research

## Abstract

In the search of animal stroke models providing translational advantages for biomedical research, pigs are large mammals with interesting brain characteristics and wide social acceptance. Compared to rodents, pigs have human-like highly gyrencephalic brains. In addition, increasingly through phylogeny, animals have more sophisticated white matter connectivity; thus, ratios of white-to-gray matter in humans and pigs are higher than in rodents. Swine models provide the opportunity to study the effect of stroke with emphasis on white matter damage and neuroanatomical changes in connectivity, and their pathophysiological correlate. In addition, the subarachnoid space surrounding the swine brain resembles that of humans. This allows the accumulation of blood and clots in subarachnoid hemorrhage models mimicking the clinical condition. The clot accumulation has been reported to mediate pathological mechanisms known to contribute to infarct progression and final damage in stroke patients. Importantly, swine allows trustworthy tracking of brain damage evolution using the same non-invasive multimodal imaging sequences used in the clinical practice. Moreover, several models of comorbidities and pathologies usually found in stroke patients have recently been established in swine. We review here ischemic and hemorrhagic stroke models reported so far in pigs. The advantages and limitations of each model are also discussed.

## 1. Introduction

Stroke is a life-threatening disease that causes neuronal loss and subsequent high rates of mortality or permanent disability. Around 17 million stroke cases occur each year worldwide, causing 6 million fatalities and leaving around 6 million patients with serious disabilities. There are two major types of strokes: The ischemic type, which is associated with the occlusion of a cerebral artery and accounts for 85% of all strokes, and the hemorrhagic stroke, which results from blood spill because of an arterial wall rupture. Clinically, stroke treatment is limited to interventions that restore blood flow in the ischemic stroke type, either pharmacologically or via mechanical thrombectomy, and only a small 15% of all stroke patients might benefit from these therapies. Many of the pathophysiological mechanisms of stroke (e.g., excitotoxicity, the core and penumbra concepts, cortical spreading depolarization, excess of free radical production, or inflammation) have been primarily identified as drivers of neuronal death in rodent models with gray matter (GM)-rich brains before being confirmed in human stroke. In contrast, the pathways that contribute to the complex pathology of neuronal soma-devoid areas of white matter (WM) following a stroke event are relatively understudied, and mostly use rodent models or perinatal hypoxic models of cerebral palsy. However, damage to the WM areas is increasingly recognized as a cause of long-term cognitive and motor disabilities in most stroke survivors. This is why research, development, and characterization of swine models of stroke, which combine both the mechanistic knowledge gained in rodent research and a greater degree of neuroanatomical and connectivity similarities with humans, may play a key role to bridge the gap from pre-clinical findings to clinical implementation.

## 2. Pig Brain to Model Human Stroke Pathophysiology

Pigs are big mammals with interesting characteristics for their use in biomedical translational research as compared to dogs and non-human primates (NHPs) [1]. There are ethical challenges associated with the fact that NHPs are highly close to humans, and canine models are not as socially accepted as other models due to their setting as companion animals in western cultures [1,2]. Conversely, due to their generalized use in the meat industry, pigs are higher-order animals that do not arise the same ethical issues as other animals do in a vast majority of the population [3].

**Figure 1:**
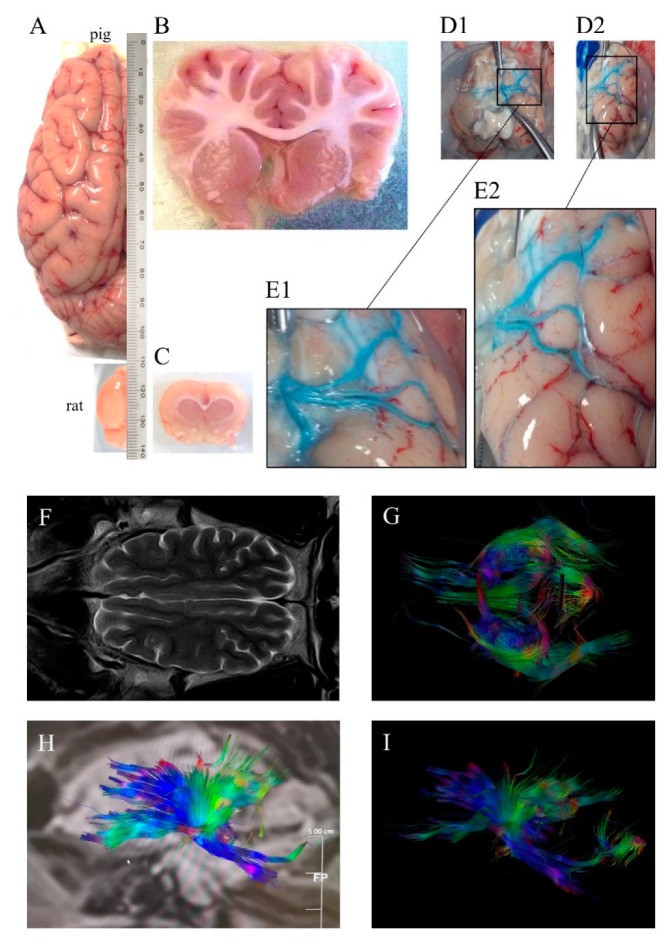
(**A**) Dorsal view of pig and rat brain hemispheres showing differences in size and gyrencephaly. Coronal (**B**) pig and (**C**) rat sections showing differences in white matter. (**D1**) Ventral view of pig brain (without cerebellum) injected unilaterally i.a. ex vivo a blue dye, (**D2**) ventrolateral view of the same brain, (**E1**) and (**E2**) magnifications of the insets in (**D1**) and (**D2**), respectively, showing three main middle cerebral arteries. (**F**) Horizontal pig brain section obtained by magnetic resonance T2 sequence, (**G**) tractography using diffusion tensor imaging (DTI) and (**H**,**I**) sagittal pig brain tractography, with colors denoting directionality. Images were obtained at the Comparative Medicine and Bioimage Centre of Catalonia (CMCiB).

In contrast with lissencephalic brains of rodents—and some commonly used NHPs like the marmoset [1,4]—pigs have human-like highly gyrencephalic brains (Figure 1), resembling lobes, gyri, and sulci/fissures of the human brain anatomy [5,6,7] with a comparable organization of motor and somatosensory areas to other mammals [8]. This resemblance with humans has also been found in several brain structures such as the limbic system, the brainstem, as well as other subcortical and diencephalic nuclei [5]. Swine brain mass is comparable to or greater than that of commonly used NHP models [5,9] with a well-developed prefrontal cortex in terms of cytoarchitecture and connectivity [10]. The development of the brain is remarkably similar to that in humans, including the myelination process [6,10,11,12], showing remarkable similarities in the resting-state networks and connectivity [13]. Due to fibrous dura mater, the increase in intracranial pressure (ICP) in large animal models of stroke is similar to that observed in humans suffering stroke [14]. Importantly, swine models allow a trustworthy tracking of brain damage evolution using the same non-invasive multimodal imaging sequences and instruments used in clinical practice [15,16] (Figure 1). Pigs also display complex individual behaviors and social interactions that can be analyzed using a number of validated tests [17,18]. Additionally, pigs have some neurovascular characteristics [19,20], including the diameter of their cerebrals vessels, which make them suitable for pre-first-in-human validation of endovascular devices or new neurosurgical techniques [15].

As a general rule, larger brains require longer fibers to connect distant cerebral areas. Across species, brain connectivity through white matter increases more rapidly than brain size [1,21]. The ratios of white-to-gray matter in humans and pigs are similar and much higher than those of mice and rats: Rodents have a WM brain composition of 10% compared to the >60% of both humans and swine [1,22,23] (Figure 1). The failure in stroke clinical trials of many neuroprotectants that are beneficial in rodents is thought to be in part due to the fact that the study of treatments with potential protection against ischemic axonal damage in the preclinical phase are seldom considered [24,25]. Hence, using larger gyrencephalic animals with a WM volume similar to that in humans could be pivotal in the preclinical study of stroke pathophysiology and of new clinically effective treatments [26,27].

Another aspect of the swine that should not be disregarded in stroke research is its size (Figure 1), as large animal species offer some advantages compared to smaller species. To begin with, depending on the strain and at sexual maturity, pigs can have a weight similar to that of humans, allowing the use of equipment and procedures specialized for humans in the clinical arena [2,3,28]. Additionally, their size allows repeated sampling of body fluids with minimal physiological disturbance, which eases performing longitudinal studies [3,29]. Moreover, pigs are much closer genetically to humans than mice [30,31], and have the potential to offer a better modeling of human gene regulation and function [32]; also, several models of pathologies and comorbidities usually found in stroke patients, such as obesity or atherosclerosis, have been established in swine [33]. The shorter the phylogenetic distance between the model and the modeled species, the better inference of the results obtained is anticipated [2]; moreover, animal models with comorbidities could potentially improve that inference. In sum, all these features make the pig an excellent research animal to obtain results with more translation to the clinic.

**Figure 2:**
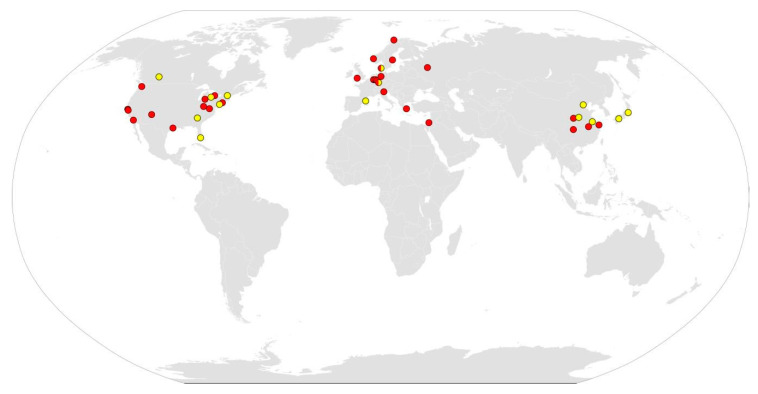
World map showing the locations where some of the main ischemic (yellow) and hemorrhagic (red) stroke models in swine mentioned in this review have been developed.

In this article we review adult swine stroke models only, not the neonatal hypoxic-ischemic encephalopathy model, as they better mimic the more prevalent human stroke pathology. In Figure 2, we show a world map with the locations where some of the main ischemic and hemorrhagic stroke models in swine mentioned in this review have been developed.

## 3. Importance of White Matter Injury in Stroke

WM injuries are normally found in elderly people in the general population [34,35]. In strokes caused by the occlusion of large cerebral vessels, WM axonal fibers are often affected [36,37,38]. WM damage with axon degeneration has been found in post-mortem human brains from ischemic stroke patients [39,40]. Additionally, lacunar stroke, a stroke subtype that involves WM damage caused by the impairment of small vessels supplying subcortical areas of the brain, accounts for 20–30% of all acute ischemic strokes [41,42]. Despite being potentially silent at the beginning, lacunar stroke causes progressive and cumulative damage, and increases the risk of stroke recurrence [42]. The presence and progression of WM injuries have been correlated with a worse clinical outcome in stroke [43,44], as well as with cognitive [37,40,45,46,47,48] and motor deficits caused by this disease [40,46,49,50].

In adult swine models of experimental ischemic stroke and intracerebral hemorrhage (ICH) significant damage and/or edema in the WM have been reported [16,27,51]. In fact, a recent study with a pig model of ischemic stroke causing deterioration of tracts from the internal capsules showed impaired motor function [16]. Moreover, a lacunar stroke pig model showed progressive WM damage, including the corticospinal tract [52]. These effects occur partly due to the differential composition of WM as compared to GM. While GM mainly consists of neuronal somas, dendrites, and axons for local signaling, supported by glial cells, WM lacks neuronal bodies and is composed of long-tract axons and the axon-supportive glial cells (singularly oligodendrocytes and fibrous astrocytes). These tracts associate different cortical areas within the same hemisphere or between hemispheres, and interconnect cortical and subcortical structures [37]. The water content is lower in the WM compared with the GM, whereas the lipid composition is higher, mostly due to myelin. Oligodendrocytes (OLs) in the CNS are in charge of wrapping axons to form segments of myelin sheath along the axon length, thus allowing the saltatory conduction of action potentials [53] and providing metabolic support to axons [54]. OL precursor cells (OPCs) are ubiquitous in the adult brain parenchyma, and proliferate to maintain a fairly constant density of mature OLs [55,56].

Most of the in vivo studies addressing the effect of ischemia on WM brain damage and its pathophysiological mechanisms have been performed in rodent models of perinatal hypoxic/ischemic cerebral palsy that do not describe the mechanisms of stroke in the adult brain. Especially in the neonatal period, the brain is consolidating the myelination process with most of the OPCs switching to myelin-synthesizing mature OLs and, thus, perinatal damage in WM results mainly from the impairment of the perinatal myelination process. Moreover, recent evidence suggests that fetal OPCs are more vulnerable than adult ones to oxygen and glucose deprivation [57].

Regarding the differential mechanisms involved in GM and WM damage, peri-infarct depolarizations and spreading depolarization have been observed to propagate in the GM but not through the WM [58]. In addition, it is well known that WM, especially deep WM, receives less blood flow and less collateral circulation compared to GM, in both humans and swine [47,59]. Despite GM has been traditionally considered to have lower tolerability to infarction in ischemic stroke patients [60,61,62], WM is found injured in most strokes, and there are studies reporting the high sensitivity to ischemia of the latter, possibly due to its reduced collateral blood supply [22,37]. In addition, differential expression of relevant receptors (e.g., glutamate receptors of NMDA or AMPA subtype, or hemoglobin receptors) in the specific cell types of GM (e.g., neurons) or WM (e.g., OLs) has an impact on their differential susceptibility to excitotoxicity in ischemic stroke [25,45,63,64,65,66,67] or to cytotoxicity by hemoglobin or heme iron molecules in hemorrhagic stroke [68,69,70].

Notwithstanding WM susceptibility to ischemic damage, this structure also has singular mechanisms of repair. There are reports of an increase of OPCs in ischemic brains of mice [71,72], maturing into OLs and migrating in response to demyelination or injury [56,60,73] to repair myelin around axons [72,74,75], restoring to some extent neurological function, but failing to reach a complete WM regeneration [76,77,78,79].

Studies of WM injury after stroke should be a research priority as they would help to develop strategies for neuroprotection and repair [80]. In fact, the Stroke Treatment Academic Industry Roundtable (STAIR) recommends that in order to obtain clinically relevant therapeutic agents, not only should these protect all the cellular components of the WM in addition to neurons in the GM, but also that their testing in large gyrencephalic animals with increased WM connectivity should be considered [26,81].

## 4. Interventions to Induce Ischemic Strokes in Pigs

According to the American Heart Association, 87% of all strokes are ischemic [82], caused mainly by the obstruction of major cerebral arteries, especially the middle cerebral artery (MCA) or its branches [83]. Typically, the study of ischemic stroke in rodent models has focused in the MCA territory [84]. The modeling of middle cerebral artery occlusion (MCAO) in larger animals has implied some additional complications. To start with, several large mammals typically used in research, in contrast to humans, have a network of small vessels at the base of the brain prior to the internal carotid artery (ICA), termed the rete mirabile, which has reportedly impeded the occlusion of intracranial vessels such as the MCA through endovascular approaches [85,86]. The attempts to circumvent this hindrance through the occlusion of the major vessels irrigating towards the rete mirabile, such as the common carotid or the pharyngeal artery, have so far rarely been successful due to the well-established collateral flow in the brain [85,87]. Additionally, compared to the single MCA in each brain hemisphere of humans, pigs have between two and up to four MCAs on each side, with anatomical variations found even within the same breeds [7,88,89] (Figure 1). Therefore, these differences between animals could complicate infarction reproducibility.

As mentioned above, for this section we excluded the neonatal piglet models of hypoxia/ischemia that have been commonly used for decades [2]. These piglet models achieve global cerebral ischemia through different mechanisms. Usually, it is performed by blocking carotid artery blood flow bilaterally, either by external compression [90] or through a surgical approach [91]. There are other strategies such as performing a generalized circulatory arrest [92,93] or obstructing brain blood flow by increasing ICP [94]. However, these approaches in neonatal or very immature piglets are more useful as models of cerebral palsy, which is caused by prenatal hypoxic-ischemic events that damage the brain’s WM and is the most frequent birth disorder [95].

Here we address the different techniques that have achieved generating partial ischemic strokes in pigs. In total, five different approaches (Table 1), including electrocoagulation, clip/ligature occlusion, endovascular embolization, photothrombosis, and endothelin-1 (ET-1) injection, have been used so far to prevent the blood supply, especially to the MCA territory, and to induce focal brain ischemia in adult/pediatric swine.

### 4.1. Electrocoagulation

Whereas the general term of electrocautery is the use of electricity to generate enough heat to destroy tissue, electrocoagulation is the use of this technique on blood vessels to achieve its permanent occlusion [138]. This method, together with microvascular clipping, was the first used to produce MCAO in swine in 2000. It was likewise the first model achieving a focal cerebral infarction reported in pigs. Sakoh et al. used a transorbital approach to reach the left MCAs, who reported that there are generally two, and occluded them proximally together with the ICA distally [111]. They used the same model in subsequent studies [112,113,114,115]. Interestingly, in one of such studies they described inter-individual differences in the model in terms of collateral blood flow to the MCA territory, and correlated differences in blood flow with the infarct size [112]. The same method has also been used by Zhang et al., also occluding the proximal MCAs unilaterally [89]. Subsequently, Imai et al. were the first to use craniotomy instead of a transorbital approach to expose the left MCAs, and then occluded their 2 branches from the origin of the lenticulostriate artery to past the olfactory tract, as well as the ICA by electrocoagulation in miniature pigs [139]. Since that first use, craniotomy has been the surgical procedure of choice to expose MCA area prior to electrocoagulation, probably to avoid potential complications related to the removal of an eyeball. Particularly, West’s group has used this swine model in multiple studies, reportedly occluding the distal MCA [16,101,102,103,104,105,106]. This method has allowed the production of consistent strokes with high survival rates in minipig strains after occluding different intracranial arteries [52,101,102,107,139].

With electrocoagulation, vessels of interest can be accurately targeted, which translates into a higher reproducibility of the focal brain ischemia models generated. On the other hand, this technique causes an irreversible occlusion of the vessels, impeding the reperfusion of the infarcted tissue and there is still the need to use invasive surgical interventions to expose intracranial vessels prior to the electrocoagulation. Particularly, eye enucleation is considered more severe due to the obvious loss in the field of vision of the animal and possible post-surgical complications as the infection of the orbital cavity [140]. However, a less invasive approach as craniotomy is also mediated by a surgical approach that could have additional effects on the brain such as increased blood-brain barrier permeability due to ICP changes [141].

### 4.2. Microvascular Clip

As mentioned above, Sakoh et al. used this technique in the first pig model of MCAO in a complementary manner to electrocoagulation. This method allowed a transient arterial occlusion as reperfusion was performed simply by removing the micro-clips occluding the target arteries [111]. As with electrocoagulation, arterial clipping also requires the exposure of the MCA surgically, which can be performed either with a transorbital approach or craniotomy. The first case of the combination of craniotomy and microvascular clipping was performed by Mattingly et al., in which only one of the 2 to 3 branches of the MCA running beneath the posterior frontal lobe was clipped for 3 h using the Imai et al. procedure, generating variable and small strokes [108]. This combination has been used in further studies to induce malignant strokes in domestic swine, showing that larger infarct volumes are generated if the occlusion of the 2 unilateral MCAs is maintained for longer periods [86]. The transorbital approach has persisted in recent papers for experimental procedures not requiring post-surgical recovery [7,116]. In this latter study, they observed the variability in the MCAs between pigs from the same breed, finding between 2 and 4 MCAs in each hemisphere, and proposed that variability of infarct size in previous studies that report the occlusion of all the MCAs could be explained by the ineffective occlusion of some of such arteries. They also suggested that the frontotemporal craniotomy to expose the MCA area, despite being less invasive, could be the reason for missing some of the MCA branches in previous studies, a problem that is not seen with the transorbital approach [7]. 

The use of microvascular clips to achieve MCAO has the added benefit of allowing the infarction to be temporary. However, the drawback of the need of surgery to allow its use is still present.

### 4.3. Endovascular Embolization

With the development of thrombolytic therapies, and particularly of thrombectomy devices, pigs have been used as models due to the anatomical and physiological similarities of their cardiovascular system with that of humans [3]. However, the objective of these studies has generally not been to generate a cerebral infarction, but to occlude extracranial vessels anatomically similar to human intracranial arteries and to study thrombolytic techniques instead [130]. The main reason is the presence of the previously mentioned rete mirabile in pigs, which has impeded reaching intracranial vessels with endovascular approaches to date [85,86].

The main method to achieve endovascular embolization of arteries in pigs has been the injection of autologous blood clots to extracranial vessels [118,119,120,121,122,123,124,125,126,127,128,129,130,131]. The embolization of an artery implies the reduction of its blood flow [142]. For instance, bilateral thromboembolism to reduce blood flow in both ascending pharyngeal arteries caused multiple and variable focal areas of stroke damage, although consistent temporo-parietal infarcts were not generated as seen by diffusion-weighted imaging [127]. Other methods of endovascular embolism used in this species are mechanical embolization [117], through diverse embolic agents including collagen microbeads [132], dimethyl sulfoxide (DMSO) [137], air [136], CO_2_ [135], Eudagrit polymer [133], and sodium alginate [134]. The use of embolic agents in pigs arose from the use of the rete mirabile as an arteriovenous malformation model. Arteriovenous malformations are an abnormal formation of blood vessels that shunt arterial blood directly into veins without passing through the capillaries. These malformations are clinically treated through endovascular embolization [142]. Nevertheless, using embolic agents as a method to produce brain ischemia in pigs has seldom been performed. Unilateral embolization of brain-irrigating arteries produced little ischemic damage to the brain. The only study that reports achieving infarcted areas in the brain this way is with the injection of sodium alginate into the ascending pharyngeal artery in Bama minipigs through the unilateral occlusion of the rete mirabile [134]. This embolization caused scattered damage throughout the brain, infarcted areas being mainly observed at the temporal and parietal lobes, and/or basal ganglia, one week after the injection.

Endovascular embolization is a minimally invasive technique used to achieve ischemia in target regions. Additionally, depending on the method used, transient ischemia with its consequent reperfusion can be performed. However, despite the study of Cui et al. [134], the presence of the rete mirabile seems to avoid the possibility of reaching intracranial vessels through an endovascular approach.

### 4.4. Photothrombosis

This method was first used in swine in 2007 as a model of acute ischemic stroke in pediatric animals, and required enucleation of the eye to reach the MCAs [88]. Since then, similar models have been generated by Armstead et al. [96,97,100,143], but to our knowledge, it has not been replicated in mature pigs and it is not as widely used as in other species such as NHPs and rats. Photothrombosis is the formation of a stable thrombus of aggregating platelets, fibrin, erythrocytes, and other blood components, due to endothelial peroxidative damage. This is generated with the photochemical reaction caused by the interaction of intravenous photosensitizing dye erythrosine B and the focused beam of a laser [88,96]. When used in pigs, photothrombosis of all the MCAs unilaterally, with reportedly 2–3 main and 1–3 smaller arteries supplying the MCA territory, produced a moderate infarct affecting both GM and WM [88].

In rodents, photothrombosis of the MCA can be generated without craniotomy due to the thinness of their cranial bones [144], differently from the markedly thick ones in pigs [28]. Thus, photothrombosis of intracranial arteries in pigs requires exposure of the target vessels by transorbital access or craniotomy [1], which implies a more invasive surgery. Overall, this model permits the occlusion of intracranial vessels with its exposure to reperfusion by using common thrombolytic methods.

### 4.5. Endothelin-1 Injection

This is the most recent model of porcine focal ischemic stroke, used for the first time by Elliott et al. and published in the year 2014 [109]. In this study, ET-1 was pumped into the cortical tissue to achieve cerebral ischemia, but infarction was not completely generated in the whole region of interest. ET-1 was firstly described as a vasoconstrictor factor present in the conditioned media of cultured bovine endothelial cells in 1985. For that reason, it was used primarily in rats as a novel method to achieve MCAO [145]. Although it was not used to cause a stroke, the effect of ET-1 on the vasoconstriction of intracranial vessels had been assessed in pigs [146] as a consequence to the observation that the levels of this molecule increased upon ICH in patients, and that this event entailed vasoconstriction in the affected area [147].

ET-1 injection is a method with which transient focal ischemia can be achieved; it is reproducible in terms of the possibility of accurate positioning of the injection and volume/concentration injected. Studies in rodents and NHPs show that infarction severity can be modulated through the concentration of injected ET-1 [23,148]. Despite it having been performed without approaches as invasive as craniotomy or eye enucleation, it still requires a surgical exposure of the target area, the common approach being through a computer tomography (CT)-guided burr hole [59,109,110], allowing the administration to virtually any cerebral region. For instance, the anatomically-driven location of the MCA through a craniotomy between the zygoma and orbit allows the occlusion of the MCA branches resulting in significant brain parenchyma lesion [89]. The use of invasive surgery might further affect the animal’s overall well-being in a stroke-independent manner. Additionally, the post-mortem analysis of human brains showed the expression of ET-1 receptors in non-endothelial cell types such as neurons [149], and the cerebral expression of ET-1 and its receptors seem to be altered in ischemic conditions [150,151]. Thus, the injection of ET-1 to induce ischemia through vasoconstriction could have unwanted effects that do not occur in the human pathology [152]. In addition, a study using this pig model reported variations between animals in the cerebral blood flow in response to ET-1 injection to the MCA territory, and in some of the animals, ischemia was not extended for enough time to generate an irreversibly infarcted region [110]. Finally, it is unclear if this model allows the control of the exact duration of ischemia and, thus, to set up reperfusion with as much control as other mechanisms of arterial occlusion.

## 5. Interventions to Induce Hemorrhagic Strokes in Pigs

Around 10–15% of all strokes are non-traumatic intracranial hemorrhages [153,154], which are classified according to the intracranial compartment in which the bleeding occurs. Within the meninges, hemorrhage can be subarachnoid, subdural, and epidural [154], the former being the most common of the three and accounting for 3% of all strokes [82]. Subdural and epidural bleeding are more typically caused by trauma [154] and the existent pig models of hemorrhage within these spaces are generally used to model traumatic brain injury [155,156] rather than spontaneously occurring hemorrhagic stroke. Most ICH events, accounting for 10% of all strokes [82], occur directly in the brain parenchyma, whereas hemorrhages into the ventricular system are less common [154]. 

The subarachnoid space of the meninges surrounding the swine brain resembles that of humans [157]. The porcine brain allows for the accumulation of larger amounts of blood and clots in subarachnoid and intracranial hemorrhage models compared to rodents due to its morphological features [153,157,158], mimicking better what happens in the clinical arena. As explained above, blood accumulates in the brain parenchyma after the onset of the bleeding, disrupting the local brain anatomy and increasing local pressure rapidly. In a second phase, compounds and blood cells entrapped in the brain parenchyma promote cytotoxicity. Accordingly, blood experimentally injected either into the subarachnoid sulcal spaces or intracerebrally has been reported to mediate cortical spreading depolarizations (CSD) in swine as well as to induce ischemic cascades and perihematomal cortical infarcts [157,159]. Importantly, swine models allow a trustworthy tracking of hemorrhage volume and brain damage evolution using the same non-invasive multimodal imaging sequences used in the clinical practice [15].

As with ischemic stroke models, we excluded neonatal piglet models of ICH in this section. However, we found it convenient to include models in immature pigs, especially considering that some techniques have only been reported in such models. These techniques include the intracerebral injection of collagenase and the more recent sonographic disruption of brain vessels (see Table 2). To better mimic specific types of hemorrhagic stroke, the injection of blood can be performed in different intracranial regions.

### 5.1. Autologous Blood Injection in Meningeal Spaces

The first studies using pigs as models of hemorrhagic strokes were performed in the 1980s, in which subarachnoid hemorrhage (SAH) was achieved through the injection of autologous blood in the pontine subarachnoid cistern after exposing it through a C-2 laminectomy [227,228,229]. Similarly to the occurrence of hemorrhagic stroke, the subarachnoid space has been the preferred target within the porcine meninges to model hemorrhagic stroke. The use of epidural injections was reported shortly thereafter [221], but this model is unusual today. The subdural space has been targeted to inject autologous blood; however, this approach is used as a traumatic brain injury model [155]. New models were developed to expose the subarachnoid space and infuse it with autologous blood to simulate SAH (Table 2). Besides the aforementioned laminectomy of the C-2, autologous blood has been injected directly to the cisterna magna, with a puncturing technique also used to obtain cerebrospinal fluid [223,224,225,226]. Likewise, craniotomy has been used to reach the MCA territory [216,217], the suprasellar subarachnoid cistern [218,219,220], and between the crests of the superior frontal and motor gyri [157]. Basal subarachnoid cisterns have also been reached through burr holes directed to the anterior skull base [214,215], in studies that exhibit the impact of transient ischemia derived from the SAH. Finally, a recent study showed that the subarachnoid space could be achieved precluding craniotomy, through the direct transorbital injection of blood into the interpeduncular cistern, without eye enucleation [213].

### 5.2. Intracerebral Autologous Blood Injection

ICH was first modeled in pigs by Farstad et al. in 1994. In this study, they injected autologous blood in the lateral ventricles of neonatal piglets [230]. An analogous technique was used in juvenile pigs in 1997 [195], but intraventricular hemorrhage models remain rarely used. The approach used to access the ventricles to inject blood has been the cannulation of the lateral ventricles through a stereotaxically- [195,196] or magnetic resonance imaging (MRI)-guided [198] cranial burr hole.

The first reported parenchymal hemorrhage porcine model using autologous blood injection was performed in piglets in 1996 [158]. In that model, the blood was slowly infused into the WM of the frontal lobe of the brain through a catheter inserted by stereotaxic surgery. The following ICH models of autologous blood injection are based on that first study. In the year 2000 the technique was refined: A balloon catheter was introduced through a cranial burr hole to generate a space in order to prevent reflux from the intraparenchymal injection of autologous blood in the frontal lobe of pigs [183]. Subsequently, the same group adapted a double-injection method from rats to pigs, together with the balloon catheter technique, to further avoid the reflux effect of the injection [172,173,176,177]. This model has also been replicated by other groups [174,175,179,180,181,182], as well as using the double injection method without the previous balloon catheter [168,170]. A similar method has been used by Bimpis et al.; in their approach a balloon is inflated and, while decompressed, the autologous blood is injected [184,185,186]. In a recent study, injection of blood to the target region of the brain was performed after a craniotomy, but this was only performed to apply focused ultrasounds to liquefy induced intracerebral blood clots [165]. However, the main method remains to be the surgical access to the target area and direct injection of the blood through a catheter or needle [158,159,187,188,189,190,191,192,193,194,197,199,200,201,202,203,204,205,206,207,208,209,210,211,212].

### 5.3. Intracerebral Collagenase Injection

The first study using collagenase as an ICH model in juvenile pigs was done by Mun-Bryce et al. in 2001 [161]. The authors injected this compound, capable of disrupting the blood-brain barrier, into the primary somatosensory cortex after exposing it through a craniotomy, and reported recurring episodes of spreading depolarization. The first in vivo use of this enzyme was performed in rats by the same group. In that study, collagenase was injected in the caudate nucleus with the aim of degrading the collagen from the basal lamina of blood vessels to cause ICH [231]. To our knowledge, only this group has used the collagenase (juvenile) swine model to induce hemorrhagic stroke in successive studies. Interestingly, in such studies Mun-Bryce et al. show a rapid depression of cortical excitability in the ipsilesional hemisphere accompanied by a gradual depression in the functionally-related contralateral region, whereas callotomized animals—lacking multiple interhemispheric axonal connections—showed a gradual increase in the excitability of the contralesional site within the acute phase. This could be indicative that the ICH damages brain areas connected to the injured region [163]. Furthermore, they observed a depressed cortical excitability in functionally-related areas of the contralesional hemisphere after this acute phase, and this impairment was preceded by a rise of the inflammatory and extracellular matrix remodeling marker MMP-9 in the ipsilesional and contralesional regions, especially in the WM [162]. However, a subsequent study reported spreading transient depolarizations during acute ICH in disperse brain areas including the contralateral hemisphere, but with no differences caused by callosotomy [164]. All these observations are indicative of the consequences of WM damage in brain connectivity caused by ICH, and the translational advantages of using the pig in neuroscience research due to the higher development of its WM. Therefore, it would be of interest to further study the WM connectivity in a collagenase adult pig model.

### 5.4. Sonographic Blood-Brain Barrier Disruption

The initial report of sonographic disruption of the blood-brain barrier in swine was performed by Aviv et al. in 2014. In this study, they targeted the vessels within the basal ganglia of young swine using MRI-guided sonography with the objective of generating a model of ICH that allowed them to quantify hematoma through the MRI detection of extravasated contrast [166]. Rupture of intracerebral vessels is thus performed with the magnetic resonance-guided focused ultrasound (MRgFUS), a technique used to perform minimally invasive targeted tissue surgeries based on the thermal ablation caused by focused ultrasounds at determined frequencies. The low invasiveness is due to both the precise ablation of the target tissue and the possibility of a transcranial approach, i.e., without removal of skull bones [232]. MRgFUS was applied in pigs by the same group in a following study to further characterize this ICH model [167]. Finally, hemorrhagic lesions were caused in the swine brain using the same technique, not to generate an ICH model per se, but to determine the non-lesional frequencies and durations of the ultrasound pulses to the brain [160]. Curiously, this technique has been used as a potential therapy for ICH in swine models. Using intracerebral autologous blood injection models in pigs, MRgFUS can be directed to the blood clots within the brain to cause its thrombolysis, showing positive results [165,198,212].

Overall, MRgFUS is a minimally invasive technique with which a targeted ICH can be precisely generated. Nonetheless, it has not been widely implemented so far, perhaps due to the lack of access to this relatively modern technology. Another problem of this technique is that its transcranial use can be intricate. Firstly, as the swine skull thickness is variable and generally big, transmission of the different ultrasound beams through this structure can make deep brain structures impossible to target. Secondly, lesions directed to regions excessively near the skull bones can cause regional overheating [232]. Actually, in the study of Xu et al. a craniotomy is performed to prevent these issues [160]. As with the collagenase model, more research needs to be performed using this promising model, although this might be hampered until a refinement of the technique is achieved.

## 6. Neurological Function Assessment in Pigs

Using in vivo models of stroke pathology gives the advantageous possibility of evaluating the neurological outcomes associated with the disease and, with more complex organisms, more detailed resemblance to the human pathology is expected. The cognition of pigs has been studied throughout the decades using different approaches. Several tasks and maze-based tests have been developed and adapted to pigs to assess distinct cognitive and behavioral aspects such as memory and learning, affective behavior, or social interactions, among others (reviewed in [18]). Despite this, there is not a current consensus about the assessment of neurological deficits in pig stroke models. In the clinical arena, there are different scales and scoring systems validated to determine neurological function, but none has been established as ideal. The first assessment performed in a stroke clinical trial was the modified Rankin scale and is the most commonly used for functional evaluation [233]. This scoring system has shown to correlate with other measures of stroke pathology as the infarct volume, as well as other neurological function scales [233,234]. In swine, the modified Rankin score has been recently adapted to the species in a study from West’s group [106], which has developed numerous studies using MCAO swine models. However, a validated scale adapted to the swine is still needed to better assess the neurological outcome. So far, the first studies that evaluated neurological function in ischemic and hemorrhagic stroke swine models adapted scales from the dog, such as Purdy et al.’s [235] and Tibbs et al.’s [236] canine stroke models.

The first neurological examination used in a pig model of focal ischemic stroke was performed by Imai et al. in 2006. They observed that permanent MCAO caused hemiparesis in the forelimbs and hindlimbs, whereas ICA occlusion caused less severe deficits with occasional hemiparesis 24 h after the surgery [139]. Subsequently, Tanaka et al. adapted a modification of the scale used for the neurologic evaluation of dogs by Tibbs et al. to their pig model of lacunar stroke, which was generated to target subcortical WM [52]. Their 25-point scoring system remains the more widely used in pig stroke models. This scale has been used in the ET-1 MCAO model of Zhang et al. [87] and—with variations—in the bipolar coagulation of the MCA model by Lau et al. from West’s group [105]. Cui et al. developed a 100-point neurological assessment scale for their ET-1 injection model, based on Purdy et al.’s system [134]. Similarly, Platt et al., also from West’s group, mention the use of a standardized neurological examination adapted from canine models to evaluate pigs after bipolar coagulation of the MCA [102]. These scales analyze different aspects of the general neurologic function of the animals including the level of consciousness (e.g., response to stimuli), motor function (e.g., limb paralysis), sensorial capacity (e.g., visual field defects), and behavior (e.g., utterance). On the other hand, a totally different approach was used almost simultaneously by the same group in the same model. In this case, they analyzed various gait parameters by video recording the animals after provoking their walk, and found a dysfunction in the limbs contralateral to the MCAO using parameters as step height (i.e., maximum height reached by the hoof when walking), swing time (i.e., time the hoof does not touch the ground when walking), and stance time (i.e., time the hoof is touching the ground when walking) [101]. This method of gait analysis has been used again recently in the same model together with the open field test to assess exploratory behavior and motor activity. In this task, the animal enters a relatively wide area and several aspects of its exploratory behavior and motor activity can be recorded by a tracking software [16,104]. Finally, the same group recently adapted the modified Rankin scale to their pig model [106].

Many of the studies with pig hemorrhagic stroke models have obviated the use of neurological tests to assess the impact of the damage generated. Rohde et al. mention that they observe the general neurological status and behavior of the pigs to rule out anomalies caused by the intracerebral autologous blood injection surgery [173]. However, it was not until 2013 that Zhu et al. used an adaptation of the previously mentioned scale from Purdy et al. to assess the evolution of neurological damage after intracerebral autologous blood injection [168], and again in 2015 [170]. Together with the latter, only the recently published article by Gerhardson et al. clearly describes the use of a scaling system to evaluate the neurological state of pigs after ICH. Specifically, they use the Tanaka et al. 25-point scale previous to and daily after the lobar WM injection of autologous blood [165].

Neurological assessment in pigs has been performed in other disease models different than stroke. The relation of such models with stroke models is that damage is caused to the brain either directly or indirectly, e.g., in models of traumatic brain injury (reviewed in [237]) or in generalized circulatory arrest [238]. Whereas circulatory arrest models have mainly used neurological scoring systems similar to stroke models, traumatic brain injury models have used varied tests and tasks to assess the impairment of different cognitive processes in pigs. One of such tests used somewhat repeatedly is the open field testing, which has also been used in the pig MCAO model [16,104,237].

## 7. Evaluation of Stroke Damage in Swine by Neuroimaging

Non-invasive in vivo imaging techniques allow a longitudinal estimation of the brain damage location and extent and contribute to our understanding of the evolution of damage after stroke. Although the extent of damage is a critical determinant, the functions served by the brain areas damaged (e.g., language, motor, or cognition) and the nature of damage (e.g., necrosis, edema, axonal damage) determine the final disabilities [239].

Magnetic resonance (MR)-based diffusion and perfusion imaging, positron emission tomography (PET), computed tomography perfusion (CTP), or a combination of them, have been used to characterize animal stroke models. These imaging techniques allow to observe parameters in the models such as post-stroke acute midline shift, which is associated with decreased survival and recovery [16,103,104,106]. Moreover, early reports allowed to assess hypoperfusion of the brain areas affected using either: (1) Apparent diffusion of water or apparent diffusion coefficient (ADC) obtained from diffusion-weighted imaging (DWI) MR combined with metabolic parameters of oxygen and glucose obtained by PET, showing that ADC below 75% of the normal tissue value indicates irreversible infarction in a transient MCAO model [113], or (2) CTP and/or PET to determine cerebral blood flow and the hypoperfusion threshold to develop infarct [59,110], or determining areas of infarct core, penumbra, or oligemia [109], as used in the swine model of induced cerebral ischemia by endothelin injection.

Conventional MRI/T2 images were used to depict infarct volume in the basal ganglia infarct model [134] and early infarct volume and neuroprotection by hypothermia [108] in an MCA model. ADC maps have been used to reveal cerebral swelling and cytotoxic edema. Reduced territorial signal intensity on ADC maps were consistently observed with the restricted diffusion that is typical of infarctions at 24 h and hydrocephalus ex vacuo at 90 days in a permanent ischemic model [101,102]. Infarct in the MCA territory is hyperintense in T2-weighted imaging (T2WI) and T2 fluid-attenuation inversion recovery (FLAIR) relative to normal GM and hyperintense in DWI [139], with corresponding hypointensity in the ADC map, indicative of cytotoxic edema [104,105]. T1, T2, and DWI hyperintensities were observed at 24 and 72 h in the ET-1 model [59,87].

WM and GM can also be accurately characterized using conventional MRI sequences as T1-weighted imaging (T1WI), T2WI, and FLAIR, based on their differences in water and lipid content. In vivo imaging tools to detect WM abnormalities, axonal loss, and demyelination are still underexplored, but new advanced MRI techniques, including quantitative susceptibility mapping (QSM) and DTI have been used recently in humans and rodents. Only a few studies have evaluated so far these MRI parameters in swine stroke. Fractional anisotropy (FA) maps obtained by DTI analysis showed loss of WM integrity in the ipsilateral hemisphere (in the internal capsule and corpus callosum) [16,240] in a pig model of stroke. In addition, a stem cell vesicle-based treatment was found to preserve the integrity and functionality of WM [103,104].When focusing on the effects of hemorrhagic stroke, leakage of blood into the brain parenchyma of swine has been mostly measured using CT [180,206,208,214] or MRI FLAIR images or in T2*-weighted images [165,173,174,178,187,213,214,241]. To overcome possible artefacts, the iron from hemoglobin can be indirectly measured using the rate of transverse magnetization relaxation, R2*(1/T2*). A recent work shows multi-contrast anatomical and quantitative parametric maps using FLAIR, T1WI, gradient echo (GRE), R2*, QSM sequences that allowed to determine the edema and hematoma areas in a swine ICH model, providing longitudinal information of the heterogeneity and evolution of the damage [191]. A dynamic contrast-enhanced (DCE) sequence allowed a real-time MRI model to study the extravasation and the acute hematoma growth [59,193]. Metabolic evaluation of the perihematoma areas by non-invasive MR spectroscopy to find salvageable tissue within the perihematoma has been recently published [193].

The imaging of other structural biomarkers, which are currently used mostly at research level, have a huge potential in the future development of personalized medicine. Precisely, characteristics of the peri-infarct structural integrity of brain around the ischemic areas as microstructural damage measured by magnetization transfer imaging (MTI) or the identification of different metabolites (e.g., glutamate/lactate/N-acetylaspartate) by MR spectroscopy will provide significant information about the pathophysiology and final outcome of stroke. Moreover, MRI signatures such as the existence of chronic old brain lesions, leukoencephalopathy, dilated perivascular spaces, microbleeds, post-stroke iron accumulation, deep infarcts, remote structural changes in WM, or cortical atrophy should be considered in swine stroke models.

## 8. Conclusions and Future Perspectives

The existing animal models of focal cerebral ischemia or ICH are only imperfect representations of human stroke. Most current stroke models are induced in healthy, adult rodents instead of in elderly individuals with comorbidities, which are the stars in human stroke pathology (for a summary of the characteristics of rodent and swine stroke models, see Table 3). Of note, the complex organization of the WM tracts that connect specific brain areas in large size brains, as swine and human brains, is not comparable with that observed in rodents. Specifically, the WM proportion in swine and human accounts for around 60%, whereas the estimation for rodents approximates 10%. Considering the differential general composition of WM compared to GM and that WM damage can be seen in the different stroke subtypes associated with clinical deficits, swine ischemic and hemorrhagic stroke models could be considered more translational due to their superior cerebral connectivity through WM and neurological behavior complexity. In preclinical studies, neuroprotection assessment has relied heavily on the evaluation of infarct volume and other short-term outcomes, preferentially studying damage and/or protection for the GM, whereas the effects on the WM connectivity or functional outcomes are usually neglected. In addition, the study of the relationship between an initial ischemia/reperfusion damage and subsequent development of hemorrhagic transformation, or of an initial hemorrhagic event and a subsequent ischemic effect in the brain neighboring areas of large gyrencephalic brains are scarce. Addressing these aspects of the pathology could be important to preclinical translatability.

The pig brain resemblance to the human brain provides a unique opportunity to study structural brain damage by non-invasive imaging techniques used in humans and to compare with acute histological and metabolic measurements not available in human studies unless in fatal cases. In addition, new gene-manipulation procedures and comorbidity models in pigs can provide in the years to come the opportunity to experimentally assess the roles of specific molecules and pathways in the pathophysiology of stroke.

The main limitations of swine brain to model human stroke are: (1) The presence of the rete mirabile, that handicaps endovascular access to intracranial arteries to produce focal brain ischemia; (2) the fact that pig plasminogen has resistance to tissue plasminogen activator (tPA)-mediated activation; (3) the significant contribution of both posterior and anterior cerebral arteries to the circulation through the circle of Willis; and that (4) pigs take longer than rodents to reach sexual maturity, adult animal models are interesting due to the relationship between ageing and the disease, but mature pigs of conventional strains can be difficult to manage considering their size. The size issue can be solved by using adult miniature pigs, which in turn are more stable strains adapted for their use in biomedical research. However, studies using aged animals are still lacking. Stroke incidence is notably affected by age and aged brains are in an enhanced inflammatory and oxidative state that renders them more vulnerable to ischemia. Concerning the rete mirabile, the study of Cui et al. mentioned above reports achieving focal cerebral ischemia by an endovascular embolization of the rete unilaterally. The replication of these results would translate into models in which a much less severe surgery is needed to induce the pathology, thus leading to a refinement of the current techniques according to the 3 Rs principle. Even so, new techniques and/or new stroke-inducing paradigms in swine (adapted from rodent stroke models, for instance clot induction by ferrous chloride or by thrombin or innovative approaches) will foster the role of porcine stroke models to bridge the gap from pre-clinical findings to clinical implementation.

## Figures and Tables

**Table 1:** Models tested to generate ischemic stroke in swine. Abbreviation; ET-1, endothelin-1; MCA, middle cerebral artery; ICA, internal carotid artery; AChA, anterior choroidal artery; APA, ascending pharyngeal artery; CCA, common carotid artery.

Type of Approach	Approach	Method	Target	Advantages	Disadvantages	References
Surgical	Craniotomy	ET-1 injection	MCA	(1) Temporary ischemia	(1) Invasive approach	[87]
				(2) Partial ischemia		
		Photothrombosis	MCA	(1) Temporary (2) Partial ischemia	(1) Invasive approach	[88,96,97,98,99,100]
				(2) Reproducible		
		Electrocoagulation	MCA	(1) Partial ischemia (2) Reproducible	(1) Invasive approach (2) Not temporary	[16,52,101,102,103,104,105,106,107]
			ICA			
			AChA			
			MCA + ICA			
		Arterial clip	MCA	(1) Temporary (2) Partial ischemia	(1) Invasive approach	[86,108]
				(3) Reproducible		
	Cranial burr hole	ET-1 injection	MCA	(1) Temporary (2) Partial ischemia	(1) Relatively invasive approach	[59,109,110]
			Striatum			
			Cortex			
	Transorbital	Electrocoagulation	MCA	(1) Partial ischemia (2) Reproducible	(1) Very invasive approach (2) Not temporary	[89,111,112,113,114,115]
		Arterial clip	MCA	(1) Temporary (2) Partial ischemia	(1) Very invasive approach	[7,111,113,116]
				(3) Reproducible		
Endovascular	Endovascular	Mechanical embolization	Extracranial arteries	(1) Minimally invasive (2) Temporary	(1) No infarction	[117]
		Blood clot injection	Extracranial arteries	(1) Minimally invasive (2) Temporary	(1) Difficult infarction	[118,119,120,121,122,123,124,125,126,127,128,129,130,131]
			CCA			
			APA			
		Polymer injection	APA-rete mirabile	(1) Minimally invasive	(1) Difficult infarction	[132,133,134]
					(2) Not temporary	
		CO_2_ injection	CCA	(1) Minimally invasive	(1) No infarction	[135]
				(2) Temporary		
		Air injection	ICA	(1) Minimally invasive	(1) No infarction	[136]
				(2) Temporary		
		DMSO injection	Rete mirabile	(1) Minimally invasive	(1) No infarction	[137]
				(2) Temporary		

**Table 2:** Models of hemorrhagic stroke generated in swine. Abbreviation; MRgFUS, magnetic resonance-guided focused ultrasound.

Type of Approach	Approach	Method	Target	Advantages	Disadvantages	References
Intracranial hemorrhage	Craniotomy	MRgFUS	Brain parenchyma	(1) Reproducible	(1) Invasive	[160]
		Collagenase injection	Brain parenchyma	(1) Reproducible	(1) Invasive	[161,162,163,164]
					(2) Only used in juvenile pigs	
		Single blood injection	Brain parenchyma	(1) Reproducible	(1) Invasive	[165]
	Transcranial	MRgFUS	Brain parenchyma	(1) Reproducible (2) Minimally invasive	(1) Target limitation	[166,167]
	Cranial burr hole	Double blood injection	Brain parenchyma	(1) Reproducible	(1) Relatively invasive	[51,69,168,169,170,171]
		Balloon catheter dilation and double blood injection	Brain parenchyma	(1) Reproducible	(1) Relatively invasive	[172,173,174,175,176,177,178,179,180,181,182]
		Balloon catheter dilation and single blood injection	Brain parenchyma	(1) Reproducible	(1) Relatively invasive	[183,184,185,186]
		Single blood injection	Ventricle	(1) Reproducible	(1) Relatively invasive	[158,159,187,188,189,190,191,192,193,194,195,196,197,198,199,200,201,202,203,204,205,206,207,208,209,210,211,212]
			Brain parenchyma			
Meningeal hemorrhage	Transorbital	Single blood injection	Subarachnoid space	(1) Reproducible (2) Minimally invasive	(1) Technically difficult	[213]
	Cranial burr hole	Single blood injection	Subarachnoid space	(1) Reproducible	(1) Relatively invasive	[214,215]
	Craniotomy	Single blood injection	Subarachnoid space	(1) Reproducible	(1) Invasive	[157,216,217,218,219,220]
		Balloon catheter dilation and single blood injection	Epidural space	(1) Reproducible	(1) Invasive	[221,222]
	Intrathecal	Single blood injection	Cisterna magna (subarachnoid space)	(1) Reproducible		[223,224,225,226]
				(2) Minimally invasive		
	Laminectomy	Single blood injection	Pontine cistern (subarachnoid space)	(1) Reproducible	(1) Invasive	[227,228,229]

**Table 3:** Characteristics of swine and rodent stroke models. Abbreviations; MCA, middle cerebral artery; ICA, internal carotid artery; NHP, non-human primate.

Characteristic	Swine	Rodents
Brain mass	80–180 g (depending on strain and age), roughly 10x smaller than human [5,9]	0.3 g (mice), roughly 4500x smaller than human; 2 g (rat), roughly 700x smaller than human [9]
Cortex morphology	Gyrencephalic [5,6,7]	Lissencephalic [1]
Myelination timeline	Similar to human, from birth to early adulthood [6,12]	Ends a few days after birth [10]
White matter proportion	60%, same as humans [1,22,23], with similar connectivity [13]	10% [1,22,23]
Intracerebral vessel diameter	Large, human-like enough to allow the use of human endovascular devices [15,20]	Much smaller, complicating surgeries [15,20]
Cerebral irrigation	High collateralization, complicates stable infarction generation [85,87], with 2–4 MCAs per side [7,88,89]. Rete mirabile to access ICA [85,86]	Lower collateralization, more stable infarction [15], with 1 MCA per side [23], and without a rete [85,86].
Dura matter	Fibrous in swine, due to brain swelling generates a human-like ICP increase [14]	Delicate in rodents, rudimentary, and underdeveloped [14]
Neurological behavior	More sophisticated and inferable to human [3]	Simpler and less generalizable to human [3]
Genetics	Shorter phylogenetic distance with human [30,31]	Extended phylogenetic distance with human [30,31]
Size	Human-like depending on strain and age, allowing multimodal imaging sequences and instruments used in human [15,16], repeated and larger biological sample collection [3,29], and procedures and equipment from humans [2,3,8]	Much smaller, different imaging instruments [15,16], limited sample extraction [3,29], and different procedures and equipment [2,3,8]
Costs	Higher [15], but lower than NHP [12]	Lower [15]
Care and use	Difficult [15]	Easier [15]
Time to sexual maturity	Prolonged, but shorter than NHP [12]	Much shorter [15]

## Abbreviations

GM	Gray matter
WM	White matter
NHP	Non-human primate
ICP	Intracranial pressure
DTI	Diffusion tensor imaging
CMCiB	Comparative Medicine and Bioimage Centre of Catalonia
ICH	Intracranial hemorrhage
OL	Oligodendrocyte
OPC	Oligodendrocyte precursor cell
STAIR	Stroke Treatment Academic Industry Roundtable
MCA	Middle cerebral artery
MCAO	Middle cerebral artery occlusion
ICA	Internal carotid artery
ET-1	Endothelin-1
AChA	Anterior choroidal artery
APA	Ascending pharyngeal artery
CCA	Common carotid artery
DMSO	Dimethyl sulfoxide
CT	Computer tomography
CSD	Cortical spreading depolarizations
SAH	Subarachnoid hemorrhage
MRgFUS	Magnetic resonance-guided focused ultrasound
MRI	Magnetic resonance imaging
MR	Magnetic resonance
PET	Positron emission tomography
CTP	Computed tomography perfusion
ADC	Apparent diffusion coefficient
DWI	Diffusion-weighted imaging
T2WI	T2-weighted imaging
FLAIR	Fluid attenuation inversion recovery
T1WI	T1-weighted imaging
QSM	Quantitative susceptibility mapping
FA	Fractional anisotropy
GRE	Gradient echo
DCE	Dynamic contrast enhanced
MTI	Magnetization transfer imaging
tPA	Tissue plasminogen activator
